# Designing an Algorithm to Preserve Privacy for Medical Record Linkage With Error-Prone Data

**DOI:** 10.2196/medinform.3090

**Published:** 2014-01-20

**Authors:** Doyel Pal, Tingting Chen, Sheng Zhong, Praveen Khethavath

**Affiliations:** ^1^Computer Science DepartmentOklahoma State UniversityStillwater, OKUnited States; ^2^State Key Laboratory for Novel Software TechnologyNanjing UniversityNanjingChina; ^3^Department of Computer Science and TechnologyNanjing UniversityNanjingChina

**Keywords:** privacy, medical record linkage, error-prone data

## Abstract

**Background:**

Linking medical records across different medical service providers is important to the enhancement of health care quality and public health surveillance. In records linkage, protecting the patients’ privacy is a primary requirement. In real-world health care databases, records may well contain errors due to various reasons such as typos. Linking the error-prone data and preserving data privacy at the same time are very difficult. Existing privacy preserving solutions for this problem are only restricted to textual data.

**Objective:**

To enable different medical service providers to link their error-prone data in a private way, our aim was to provide a holistic solution by designing and developing a medical record linkage system for medical service providers.

**Methods:**

To initiate a record linkage, one provider selects one of its collaborators in the Connection Management Module, chooses some attributes of the database to be matched, and establishes the connection with the collaborator after the negotiation. In the Data Matching Module, for error-free data, our solution offered two different choices for cryptographic schemes. For error-prone numerical data, we proposed a newly designed privacy preserving linking algorithm named the Error-Tolerant Linking Algorithm, that allows the error-prone data to be correctly matched if the distance between the two records is below a threshold.

**Results:**

We designed and developed a comprehensive and user-friendly software system that provides privacy preserving record linkage functions for medical service providers, which meets the regulation of Health Insurance Portability and Accountability Act. It does not require a third party and it is secure in that neither entity can learn the records in the other’s database. Moreover, our novel Error-Tolerant Linking Algorithm implemented in this software can work well with error-prone numerical data. We theoretically proved the correctness and security of our Error-Tolerant Linking Algorithm. We have also fully implemented the software. The experimental results showed that it is reliable and efficient. The design of our software is open so that the existing textual matching methods can be easily integrated into the system.

**Conclusions:**

Designing algorithms to enable medical records linkage for error-prone numerical data and protect data privacy at the same time is difficult. Our proposed solution does not need a trusted third party and is secure in that in the linking process, neither entity can learn the records in the other’s database.

## Introduction

### Electronic Patient Records

With the popularity of electronic patient records and the expanded use of medical information systems [1], nowadays many different health care providers store medical records of patients electronically. In many cases different health care providers hold the same patient’s data. To enhance the quality of health care treatment, for example, in regional health information networks, often it is required to gather information about the same patient from different providers [2]. In order to identify whether a particular patient’s information is held by more than one health care provider or not, a matching technique is used on the key attributes of the patient’s demographic information [2]. As another example, public health surveillance often requires linking patient records from different health care providers [1]. In order to monitor the quality of health care treatment provided in a region and to analyze a patient’s medication interaction, it is very helpful to collect correlated data from different sources [3], (eg, clinics, pharmacy, laboratory, and health care providers).

### Keeping Patient Information Secure and Private

With the increasing needs of keeping and linking electronic patient records, it is very challenging to maintain security and preserve privacy. Under the regulations of the Health Insurance Portability and Accountability Act (HIPAA) [4], preserving patient’s privacy is important in linking the patient’s data. As medical databases contain different identifiers of a patient (eg, patient’s name, surname, date of birth, Social Security Number-SSN, contact number, address, etc), using these identifiers in their actual form for linking purpose violates privacy. Moreover, due to privacy, security, and business concerns, different health care providers may not be willing to reveal their health data information other than the linking result to the other provider. Among existing research works, Shapiro et al and Vest [1,2] illustrated some approaches toward health information exchange. One obvious approach is to link data using the identifiers in encrypted format [5-8]. An elegant approach to encrypt identifiers is using one-way hash functions as in Quantin et al and Quantin et al [7,8]. To ensure security, these methods are based on the irreversible transformation property of one-way hash functions on identification data. These methods are vulnerable to some common cryptographic attacks. In Quantin et al [8], the authors proposed a computerized hash encoding and anonymous record linkage procedure on medical information. To consolidate security against dictionary attack, Quantin et al [8] used two pads, one for the sender and the information and the other one for the recipient. Some other approaches have been proposed regarding privacy preserving medical records linkage algorithms [9,10]. A trusted third party has been used in Churches and Christen [10], to make the algorithm more secure. Here each party is involved in computing the set of bigrams for each string. Each party exchanges the power set of encrypted bigrams with the trusted third party and then string similarity is performed using the Dice coefficient. However, these approaches usually have high false negative rates. Using indirect pseudonym identifiers [9], besides giving the patients control over what information is revealed about them, an architecture has been proposed to link medical records.

Some algorithms on privacy preserving data matching are proposed in database and data mining research fields. In Lindell and Pinkas [11], the authors have proposed a solution where two parties can run a data mining algorithm on the union of their own confidential databases, without revealing any unnecessary information. In this particular solution, the authors focused on the problem of decision tree learning, as the input sizes of data mining algorithms are huge and the data mining algorithms themselves are very complex. At each party’s end, this method uses a computation of the same order as computing the Iterative Dichotomiser 3 algorithm on its own databases. It combines the result using cryptographic tools. Some solutions of privacy preserving record linkage are based on the perturbed information. For example, Agrawal and Srikant [12] used a randomizing function such as the Gaussian function or uniform perturbations to perturb the sensitive data and build a decision tree classifier from these perturbed data. This solution offers a reconstruction procedure to accurately estimate the original data value distribution. The cryptographic technique, which relies on the secure multi-party computation (SMC) Protocol [13], computes functions over private inputs. Scannapieco et al [14] proposed a more efficient protocol based on cryptographic techniques, which preserves privacy of database schemas. Here, the authors consider the scenario that two parties want to link their data in string format and can have different schemas. They propose a protocol that consists of data matching and schema matching protocol. The protocol builds an embedding space and two parties embed their data strings using a variant of Sparse Map [15] ensuring the contractiveness of the embedding. A semihonest third party collects the embedded strings from the two parties and computes the similarities. Whereas Agrawal and Srikant [12] concentrated on exact matching, Scannapieco et al [14] focused on approximate matching. A hybrid method that combines both the data perturbation and cryptographic techniques is presented in Inan et al [16]. The basic idea of this method is to first classify the data into two classes as matches and mismatches, and then apply the general SMC protocol to compute the distance for the records in the matches’ class. A querying party is introduced to provide the classifier that determines matching record pairs. The problem with this method is that general SMC protocols are costly to use in practice. In Scannapieco et al and Inan et al [14,16], the proposed solutions used a trusted third party, which is a major issue since the Web-based trusted third party may not be a good choice to link records in a privacy preserving way.

Some recent works [17-20] focused on security and privacy in biological services and in medical data. By secure encapsulation and publishing of bioinformatics software in a cloud computing environment, Zhang et al [17] have derived a prototype system of the biological cloud. While they worked on only biological services and focused on achieving a prototype system of the biological cloud, our solution works on different databases and concentrates on linking these different databases in a privacy preserving manner. In Gkoulalas-Divanis and Loukides [18], the authors have discussed medical data sharing by preserving privacy and data utility. Here they have given a clear picture about the data that generally is used for data sharing purposes, different techniques for privacy preserving data sharing, and different types of threats. A new algorithm has been proposed in Mohammed et al [20] for heterogeneous health data sharing in privacy preserving manner. The proposed algorithm considers health data containing both relational and set-valued data and accomplishes “element-of” differential privacy. In Gkoulalas-Divanis and Loukides [18], the authors have discussed different types of medical data, for example, demographics, clinical information, text, and genomic information, and Mohammed et al [20] worked on heterogeneous medical data. In our solution, instead of different categories of medical data and heterogeneous health data, we concentrate on textual and numeric data and further categorize them into error-free data and error-prone data. Kum et al [19] focused on privacy preserving interactive record linkage. The authors have given a solution by proposing a computer-based third party record linkage platform, Secure Decoupled Linkage. The proposed solution decouples the data, obfuscates it, and shares minimum information via encryption, chaffing, and recoding respectively, to ensure the protection against attribute disclosure. A new computer-based third party record linkage platform has been proposed in Kum et al [19], but our proposed solution does not need a trusted third party.

However, when we consider the real life scenario, it is possible that existing works might not meet all the requirements of medical record linkage all the time in practice. For instance, earlier researches [5] on data record linkage (ie, sending identifiers in encrypted format does not allow any kind of error in identifiers) may happen frequently in real cases. Spelling mistakes and typographical errors are very common in databases. Some researches [21-24] have been done toward the error-prone data and on the missing data. In Weber et al [24], the authors have proposed a solution to build cross-site records and link data for a particular patient as he/ she moves between participating sites. They considered the hypothesis that most variation in names occurs after the first two letters; this, along with the date of birth, is one of the most reliable attributes. Out of this consideration they generated the composite identifier based on the real identifiers in such a way that the possibility of identifying a common patient is maximized. This composite identifier is the hashed string of the first two letters of the patient’s first name and last name, plus their date of birth. Considering this composite identifier, they have shown that it has a higher sensitivity rate compared to other identifiers (eg, SSN and identifier based on patient’s full name and date of birth).

Most of the existing algorithms for error-prone data are concentrated on textual data. They are very useful for linking records for any customer identifying information. Some approaches toward error-prone data in privacy preserving record linkage have been proposed. One of these proposed solutions is using Bloom filters [21]. This solution applies Bloom filters with keyed hash message authentication codes on q-grams (for a particular string, q-grams gives all possible sub-strings of length q) of identifiers and allows errors in identifiers. Compared to other privacy preserving record linkage methods with encrypted string type identifiers, these methods have lower false negative rates. However, the existing proposed solutions of this category are designed for textual data. On the other hand, privacy preserving record linkage for error-prone numeric data is also very important. For instance, medical records usually contain ample numerical attributes, such as the patient’s blood pressure, height, weight, and other test results. In different medical databases, medical data may be stored in different precisions. Then even two very close numbers (eg, 392.1 and 392.11) may cause a totally negative linking result. The consequence of high false negative results may be very harmful, especially when querying a patient’s records for emergency treatments.

### Aim of the Study

**Figure 1:**
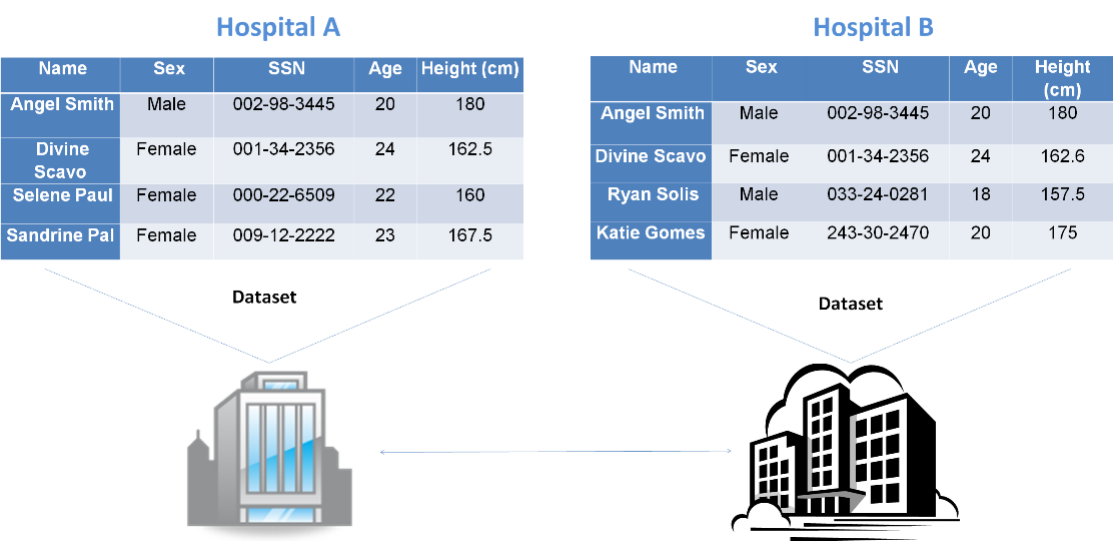
Privacy preserving record linkage problem.

In this paper we aim to address the privacy preserving record linkage problem with the presence of error-prone data. In Figure 1 we illustrate an example of real-world cases where privacy preserving record linkage for error-prone data is needed. In this figure, each of the two hospitals holds a database of patients’ information of its own. They would like to find out the common patients they share (eg, *Angel Smith* and *Divine Scavo*) in order to perform collaborative research on the shared data. However, due to the requirement of HIPAA, they cannot exchange data in clear texts. Moreover, we notice that for the patient *Divine Scavo*, all attributes are the same at both hospitals except the height (one is 162.5 cm and the other is 162.6 cm). If traditional cryptographic schemes are used, the record belonging to the same patient will be labeled as a mismatch, leading to an error result. In order to avoid the mismatch for the records belonging to the same patient, we need new software to enable privacy preserving record linkage for error-prone data.

In this paper, we designed and developed comprehensive record linkage software for medical organizations, which meets the regulation of HIPAA. Our solution for the privacy preserving record linkage will work not only for error-free data, but also for error-prone numerical data, which is never enabled in existing solutions. The design of our software is open so that the existing textual matching methods (eg, Weber et al) [24] can be easily integrated into the system. Our algorithm used in the software is correct, secure, and efficient. Furthermore, our solution does not need a trusted third party for any of the offered cryptographic schemes. This is important because in many cases such a trusted third party can hardly be found, especially when the health care providers are from different regions or even countries. With a trusted third party added to the software we can use public key cryptographic schemes [25,26]. In particular, we allow the software users to select one of their collaborators who is also using our record linkage software, choose some particular attributes of the database to be matched, and establish the connection with the collaborator after the negotiation. For error-free data, our solution offers two different choices for cryptographic schemes (ie, Secure Hash Algorithm-SHA-1 and SHA-2) [27]. For error-prone data, we propose a new linking algorithm named the Error-Tolerant Linking Algorithm. The Error-Tolerant Linking Algorithm matches the error-prone numerical data and preserves the data privacy for each user too. To achieve this goal, we securely measure the Euclidean distance between two records. If the distance is below a threshold, we say that there is a link between the two records. It is challenging to compute the distance between two records in a privacy preserving way, such that the other party can learn no information of the original attribute values. To overcome this difficulty, we carefully designed a novel algorithm utilizing the homomorphic property of an efficient cryptographic scheme, the ElGamal, in its extended form. After the linkage process, our solution is also capable of managing the matching records from recent history.

## Methods

### Design Consideration

This section describes the design consideration of privacy preserving record linkage in general, and the design consideration of record linkage for the error-prone numeric data formally in details. Preserving privacy is a real issue when two or more organizations are willing to share part of their entire data without revealing any sensitive information about any entity to each other. Assume the privacy preserving record linkage takes place between two medical organizations. Each organization holds information about its entities (eg, patients/customers). Along with the different entities, both of these organizations have some common entities too. It is very difficult to get the data of only these common entities from the entire dataset of two organizations while preserving privacy at the same time.

**Figure 2:**
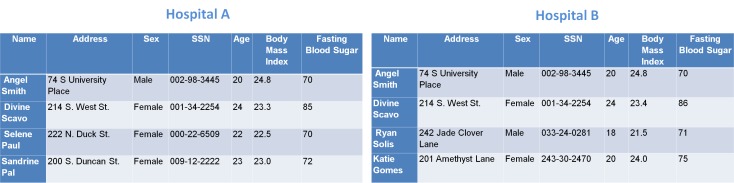
Data from two hospitals.

We can explain the overall problem as a real life scenario. For example, suppose privacy preserving record linkage takes place between two hospitals (eg, Hospital A and Hospital B). Figure 2 shows the detailed information/attributes about patients, such as, patient’s name, date of birth, address, SSN, sex, etc that each of these hospitals maintains. Hospital A has four patients, *Angel Smith, Divine Scavo, Selene Paul,* and *Sandrine Pal,* and Hospital B has four patients, *Angel Smith, Divine Scavo, Ryan Solis,* and *Katie Gomes*. All the information of patient *Angel Smith* in Hospital A matches with the patient *Angel Smith* in Hospital B. Some of the information (ie, name, address, SSN, age, and sex) of patient *Divine Scavo* in Hospital A matches with patient *Divine Scavo* in Hospital B. Assume that Hospital A is the initiated organization (ie, it takes the initiative of record linkage) and Hospital B is the participating organization as it participates in the record linkage. We use the terms initiated organization and participating organization afterwards in this paper. During this entire procedure it is implicit that Hospital B agrees to share its patients’ database with Hospital A without revealing any sensitive information about the patients. Now Hospital A should get *Angel Smith* as matched data, *Divine Scavo* as partially matched data, and *Selene Paul* and *Sandrine Pal* as mismatched data as a result. Note that, here matched data means the data that belongs to Hospital A as well as to Hospital B, mismatched data means data that belongs to Hospital A, but not to Hospital B, and partially matched data means data for an entity of which some of the attributes match at both hospitals’ end.

**Figure 3:**
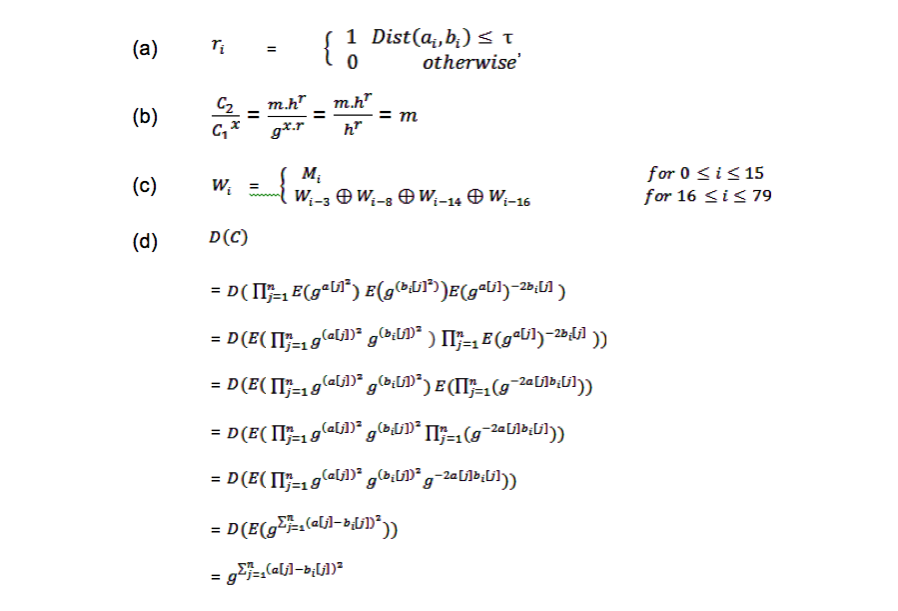
Equations (a) Output Vector, (b) Decryption equation of ElGamal scheme, (c) The expanded message of SHA-1, and (d) Proof of correctness of Error-Tolerant Linking Algorithm.

Errors in database data are very usual. Therefore privacy preserving record linkage for error-prone data is necessary too. For error-prone numerical data we can formulate the problem as follows. We assume that for any client, the common part of their records stored in both entities has *n* attributes. The goal of the linkage is to find out the records held by party B that are within a small distance (very close) to the records held by party A. Formally, the problem for error-prone data (ie, privacy preserving error-tolerant linkage) in this paper can be defined as follows–given two databases *D*_*A*_ (*a*_1_, *a*_2_, … . . , *a*_*m*_) and *D*_*B*_ *(b*_1_ *, b*_*2*_ *, … .* . , *b*_*m*_ *)* with the same attributes. The error-tolerant linkage function takes a tuple <a, D_B_, τ> as input, where *a* is any record in *D*_*A*_ and **τ** is the distance threshold. It outputs a vector of Boolean numbers, (r_1_, r_2_, . . ., r_m_), where ∀*i* s.t., 1 ≤ *i≤m* (Figure 3a shows the output vector), in which *Dist* () is the distance function defined for input records (in this paper we use Euclidean distance). Privacy preserving error-tolerant linkage guarantees that computing the error-tolerant linkage function is secure in the semihonest model [28,29], without a trusted third party.

By being secure in the semihonest model, we mean that the two parties (or any other adversary) cannot efficiently obtain more information than the input and the output of the algorithm. In particular, for our error-tolerant linking algorithm, the two parties will know only the output (r_1_, r_2_, . . ., r_m_) and no information about the values of records (either linked or not-linked) will be revealed.

### Privacy Preserving Record Linkage Schemes

In this subsection, we discuss the overall solution for the design consideration described in the previous section and schemes of the solution in details. The main idea is that if the participating organization sends the entire dataset as encrypted format to the initiated organization, then it is not possible for any other third party, as well as the initiated organization, to know about the real data of the participating organization if the key-pair is unknown. In our proposed solution, to send data confidentially to the other party we have considered three cryptographic schemes: (1) SHA-1, (2) SHA-2, and (3) Error-Tolerant Linking Algorithm. Before discussing the schemes in detail, we categorize the data in two different data categories: (1) the error-free data, and (2) the error-prone data. Among the above-mentioned three cryptographic schemes, the first two (ie, SHA-1 and SHA-2) are the basic cryptographic schemes for privacy preserving error-free data linkage and the Error-Tolerant Linking Algorithm is for the error-prone data.

**Figure 4:**
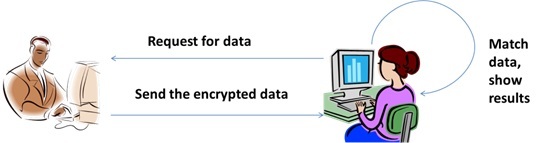
Overall flow of the solution.

The overall flow of running the system is as follows. To encrypt the data, the initiated organization chooses a dataset name and the cryptographic scheme, and sends both of them to the participating organization. If the participating organization holds the same dataset, it starts the privacy preserving data linkage process by sending the data in cipher text format to the initiated organization. Meanwhile, the initiated organization encrypts its own dataset by using the cryptographic scheme. After receiving the data, the initiated organization applies the privacy preserving matching scheme to obtain the results (ie, the matched, mismatched, and partially matched data). Figure 4 shows the diagram of the overall flow of the solution.

We will discuss the Error-Tolerant Linking Algorithm in detail, and the two existing cryptographic schemes briefly in the following two subsections, respectively.

### Scheme for Error-Prone Data

Our proposed new solution for error-prone data, as above-mentioned, is the Error-Tolerant Linking Algorithm. The Error-Tolerant Linking Algorithm uses the ElGamal [26] scheme as the basic building block. In this subsection, we review the ElGamal scheme first and then will describe the Error-Tolerant Linking Algorithm in detail. The ElGamal is a public key encryption scheme. Let *G* be a cyclic group of prime order *p* with generator *g*. A value *x* ∈ *Z*_*p*_ is randomly chosen as the private key. The corresponding public key is (p, g, h), where h=*g* ^*x*^. To encrypt the message *m*, a value r ∈ *Z* ^*p*^ is randomly chosen. Then the cipher text is *E*(*m*) = (*C*_1_, *C*_2_)=(*g* ^*r*^, *m*.*h* ^*r*^). We use *E*(*m*) in this paper to denote the cipher text of *m* encrypted by the ElGamal scheme. The decryption equation of ElGamal scheme is shown in Figure 3b.

Difficulty of computing discrete logarithms over finite fields forms the basis for security in the ElGamal. To decrypt a cipher text, any adversary would have to get the one time random integer. Determining this random integer is infeasible, as it requires computing of discrete logarithms.

The Error-Tolerant Linking Algorithm exploits the homomorphic property of the ElGamal scheme. That is, for two messages *m*_1_ and *m*_2_, it satisfies the following property,

*E*(*m*_1_.*m*_2_)=*E*(*m*_1_).*E*(*m*_2_) (1)

In addition to linking the data from two different organizations, the Error-Tolerant Linking Algorithm preserves privacy as well. We assume that the attributes of records are preprocessed and converted to integers beforehand. For numerical attributes, this preprocessing is straightforward by normalizing the original values to integers within a certain range. For attributes consisting of strings, we can use a preprocessing method to convert the strings into integers so that the integers can still keep the distance between the records. Then our algorithm can be applied afterwards to complete the records linkage. This algorithm allows the input record with minor deviations less than a small threshold τ. The threshold value is to calculate the distance between the identifiers of two records. In this algorithm, neither entity can learn the records of each other’s patients.

**Figure 5:**
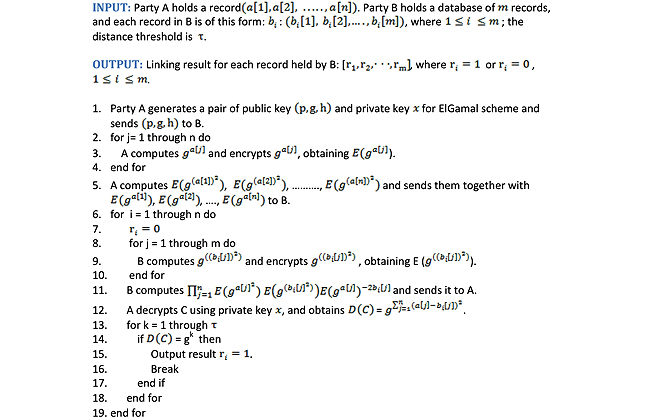
Algorithm 1: Error-tolerant linking algorithm.

Algorithm 1 shows the details of our privacy preserving Error-Tolerant Linking Algorithm. First, party A generates a pair of keys for the ElGamal scheme and sends the public key to party B. For each attribute *a*[*j*] in the record, party A computes *g* ^a[j]^ and *g* ^((a[j])^2^)^, and sends the cipher texts of these terms to party B. For each record *b*_*i*_ held by party B, party B computes *g* ^((bi[j])^2^)^ for each attribute *b*_*i*_[*j*], and encrypts them using the public key received from party A. Then party B computes C as shown in line 11 in Algorithm 1. After receiving the product from party B*,* party A decrypts it using the private key and obtains a decrypted value *D*(*C*). If *D*(*C*) equals any number in (*g* ^0^, *g* ^1^, *g* ^2^,… … ., *g* ^τ^ ), then it means that ∑^n^_k=1_(*a*[*k*]-*b*_*i*_[*k*])^2^ ≤τ, and thus we say it is a linking case. Otherwise, we say record *b*_*i*_ does not link to *a*. The Error-Tolerant Linking Algorithm is correct. We discuss the correctness analysis of the Error-Tolerant Linking Algorithm in the subsection named Correctness Analysis. Figure 5 shows the Error-Tolerant Linking Algorithm–Algorithm 1.

### Schemes for Error-Free Data

For the regular error-free data, we have considered two existing basic cryptographic schemes: (1) SHA-1, and (2) SHA-2. The SHA-1 [27] and SHA-2 [27] come under the hash algorithm family. In this subsection, we will review the SHA briefly. The SHA is based on the design principle of the Message Digest Algorithm 4 (MD4) [29]. Both of these algorithms are iterative and one-way hash functions. The SHA-1 and SHA-2 consist of two major steps: (1) preprocessing, and (2) hash computation. In the preprocessing step, every input message is padded and then split into fixed size message blocks, and this step also initializes the working variables to be used in hash computation. The hash computation consists of an 80-step compression function that iteratively generates hash values *h*_*i*_ (ie, the *i* ^*th*^ hash value). The 80-step compression function is applied to each of the message blocks. Generally, two types of inputs are considered here: (1) chaining input, and (2) message. If the message is *m* and chaining input is *h*_*i*_, then the compression function is *g*(*m*, *h*_*i*_) at the *i* ^*th*^ stage. The chaining input *h*_(i+1)_ at the (*i*+1)^*th*^ stage is calculated by *h*_*i*_+*g*(*m*, *h*_*i*_). The value of the compression function at the last stage is the hash value of the message. The SHA-1 and SHA-2 differ in terms of the message size, block size, word size, and message digest size as given in Table 1.

In the SHA-1, five working variables are used: (1) *a,* (2) *b,* (3) *c,* (4) *d,* and (5) *e*. The message is represented by 16 32-bit words, denoted by *M*_*i*_. The message is then expanded to 80 32-bit words *W*_*i*_. The expanded message *W*_*(i)*_ is shown in Figure 3c.

After that it initializes the working variables and computes the 80-step compression function and intermediate hash values. If there are *n* message blocks (ie, *M*_1_, *M*_2_, … . ., *M*_*n*_), then the entire procedure is repeated for *n* number of times. The resulting 160-bit message digest of the message *M* is,

*H*_*0*_^(N)^||*H*_1_^(N)^ ||*H*_2_^(N)^ || *H*_3_^(N)^ || *H*_4_^(N)^ (2)

Here, *H*_*j*_^(i)^ means the *j* ^*th*^ word of *i* ^*th*^ hash value.

The procedure of the SHA-2 is similar to the SHA-1. It first pads the message and divides it into 64-bit message blocks. The number of working variables here are eight (ie, *a*, *b*, *c*, *d*, *e*, *f*, *g*, and *h*). After initializing the working variables and computing the 80-step compression function and intermediate hash values, it generates 512-bit message digest of the message *M*. The final message digest of *M* is,

*H*_*0*_^(N)^||*H*_1_^(N)^ ||*H*_2_^(N)^ || *H*_3_^(N)^ || *H*_4_^(N)^ ||*H*_5_^(N)^ ||*H*_6_^(N)^ ||*H*_7_^(N)^ (3)

The SHA-1 and SHA-2 are considered here since it is easy to compute the hash value of any given message and they are the one-way hash functions (ie, they have one-way, second preimage resistant, and collision resistant properties). The SHA-1 and SHA-2 produce 160-bit and 512-bit hash values, respectively, for any given message. Therefore, for any given message, there are 2^160^ and 2^512^ possible hash values. It is very difficult to identify the actual message from this vast range of hash values [30]. Here in our system, after applying the SHA-1 and SHA-2 on the data, we get the message digest/encrypted data from both of the parties and apply data matching techniques on those encrypted data.

**Table 1:** The SHA-1 and SHA-2 properties.

Secure hash algorithm name	Message size (bits)	Block size (bits)	Word size (bits)	Message digest size (bits)
SHA-1	<2^64^	512	32	160
SHA-2	<2^128^	1024	64	512

### System Analysis

We analyze our schemes, especially the Error-Tolerant Linking Algorithm, in terms of correctness, privacy, and complexity.

### Correctness Analysis

For the proof of correctness, if the two parties follow Algorithm 1, they will jointly compute the correct Euclidean distance without each party knowing the record from the other party. The homomorphic property of the ElGamal scheme helps to prove line 12 in Algorithm 1. (Figure 3d shows the proof of correctness of Error-Tolerant Linking Algorithm.

If (*C*)=*g* ^*k*^, where *k* ≤ τ, it means that ∑^n^_k=1_(*a*[*k*]-*b*_*i*_[*k*])^2^≤τ. Then we can say that record *a* is within the distance of τ, from record *b*_*i*_, and the result of error-tolerant linking is positive.

### Privacy Analysis

In this subsection, we explain why the Error-Tolerant Linking Algorithm is secure (ie, privacy preserving) in the semihonest model. Being secure in the semihonest model means neither of the two parties can learn more than the output of the algorithm from the information received during the algorithm. In Algorithm 1, the only message received by B is the cipher texts E(g^((a[1])^2^)^), E(g^((a[2])^2^)^),…, E(g^((a[n])^2^)^), and E(g^a[1]^), E(g^a[2]^),…, E(g^a[n]^). Since the ElGamal scheme is semantically secure under the decisional Diffie-Hellman assumption [31], party B cannot learn anything about g^((a[1])^2^)^, g^((a[2])^2^)^,…, g^((a[n])^2^)^, g^a[1]^, g^a[2]^,…, g^a[n]^ but the cipher texts. For party A, the message it receives from party B is C. From the semantic security of the ElGamal scheme, party A cannot learn the clear texts from party B but the *D*(*C*). Here we note that *D*(*C*) and ∑^n^_k=1_(*a*[*k*]-*b*_*i*_[*k*])^2^ can be derived from the output of the algorithm by trying different numbers in a small range of **τ**. Therefore, we say that party A knowing *D*(*C*) and ∑^n^_k=1_(*a*[*k*]-*b*_*i*_[*k*])^2^ does not violate the security requirement and party A can send these values to party B if needed.

### Complexity Analysis

We analyze the computation cost of our algorithm on party A and party B respectively. The computation cost of party A includes computing 2*n* exponentiations, 2*n* encryptions, and 1 decryption. Suppose that each exponentiation takes time *T*_*e*_. Then the total computation cost of party A is 2*n*(*T*_*e*_+*T*_*E*_) +*T*_*D*_, where *T*_*E*_ is the time to perform one ElGamal encryption and *T*_*D*_ is the time to perform one decryption. Values of *g* ^*k*^ can be computed beforehand and saved in a table for reference at line 14, Algorithm 1. Party B needs to compute 2*nm* exponentiations, *nm* encryptions, and 2*nm* divisions/multiplications. So the computation cost for party B is *nm* (2*T*_*e*_+*T*_*E*_+2*T*_*m*_), where *T*_*m*_ is time for a multiplication, and the definitions of *T*_*e*_, *T*_*E*_ are as above. When there are *k* records in party A to be linked with the records held by party B*,* the total computation time for party A is *nm* (2*T*_*e*_+*T*_*E*_)+2*nmk* × *T*_*m*_. Note that in the computation cost, *nm* (2*T*_*e*_+*T*_*E*_) is not multiplied by *k*, because as long as party A does not change their public key, the cipher texts of party B’s records do not change, and thus they only need to be computed once.

## Results

### System Implementation

This subsection explains the system implementation we have taken into account for the problem described before. We implement our system using the Eclipse Integrated Development Environment. We have used the programming language Java. The entire system is divided into three modules: (1) Connection Management Module, (2) Data Matching Module, and (3) Matching Record Management Module. Among these three modules, the main module is Data Matching Module. The solution of the privacy preserving record linkage (ie, Data Matching Module) works for both the error-free data and error-prone numerical data. The Matching Record Management Module shows the result/records from the recent past data matching attempts, and the Connection Management Module takes care of creating a connection with the collaborator. We will discuss these three modules in detail in the following subsections. For the snapshot of selecting a function/module in our system, see Multimedia Appendix 1.

### Connection Management Module

Each party/organization keeps a list of available and reachable collaborators. To create a connection with another party/collaborator, each party needs to select that particular collaborator from the collaborator list. For the snapshot of how a user selects a collaborator in our system see Multimedia Appendix 2. To initialize the connection, each and every organization keeps some initial information about the other collaborators beforehand. This information contains the Internet Protocol (IP) address, port number, public-key, private-key pair, etc. Party A first selects party B from the available participating collaborator list. Party A uses the corresponding IP address and port number of party B for creating a connection. We follow the client-server architecture to implement our system. The communication between two parties is realized by socket application program interface (API). Party B (server) creates a socket to listen to requests from party A (client). Party B can handle more than one client at a time. In that case, party B creates a separate socket for each of the requesting clients using multi-threading. To be precise, a user can work as both a client and a server at the same time. A user can turn on the server and continue working as a client using the data matching procedure.

### Data Matching Module

**Figure 6:**
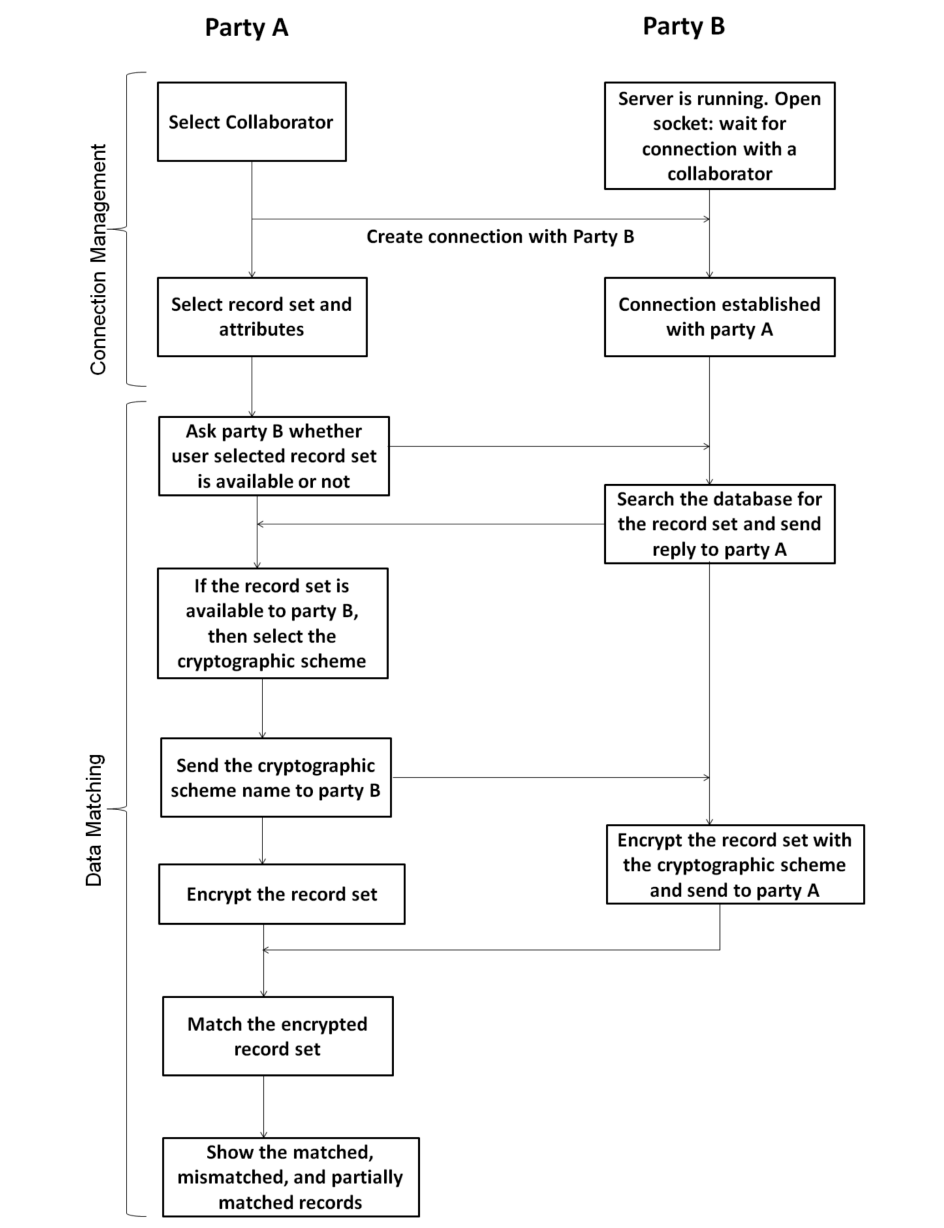
The workflow of the system.

The Data Matching Module is the main module of our system. To explain this module, we consider there are two parties: (1) party A, and (2) party B. Party A initiates the matching procedure and party B takes part in this matching procedure. Figure 6 shows the entire workflow of the system including party A and party B.

**Figure 7:**
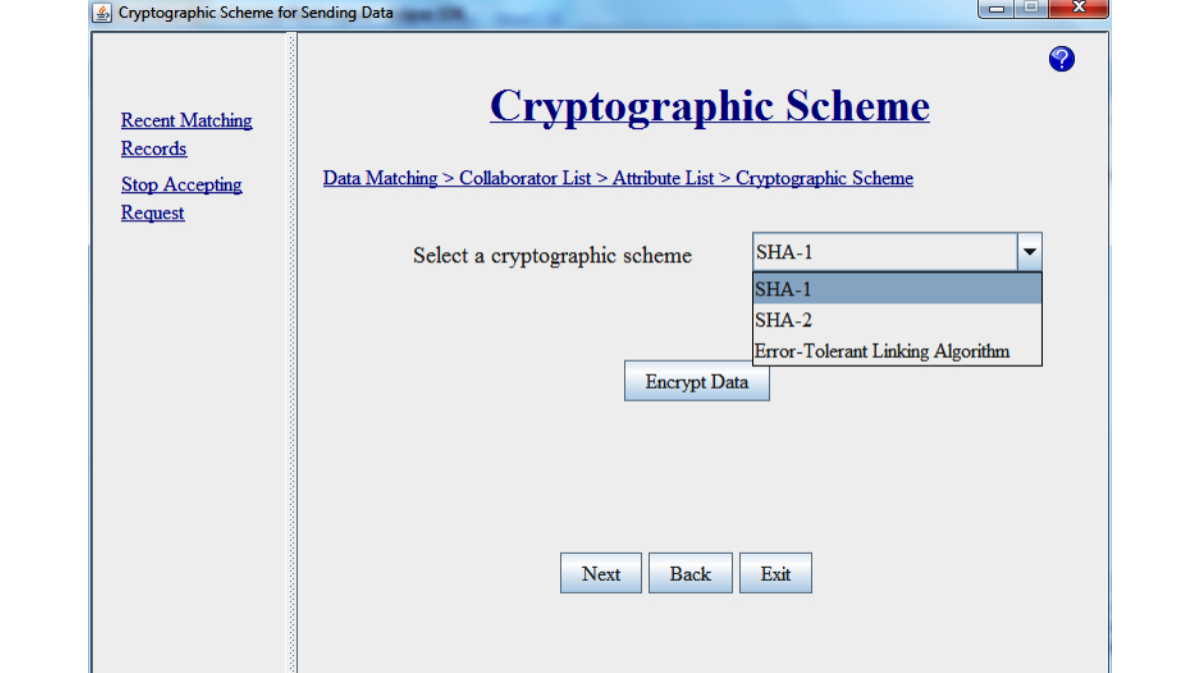
Snapshot of selecting a cryptographic scheme.

As shown in Figure 6, once a connection is created between party A and party B, data transfer between the two parties and matching can take place. Party A first selects the record set for matching data. When party A selects the dataset name, then the corresponding attributes’ list becomes available. Party A selects the attributes’ names and sends the dataset name along with the attributes to party B. Party B searches the requested record set in its set of record sets. If party B has the record set, it sends the acknowledgement to party A. In response to this acknowledgement, party A sends the user selected cryptographic scheme name to party B and encrypts its own selected record set. Party B encrypts the requested dataset with the requested cryptographic scheme. Figure 7 shows the snapshot of how a user selects a cryptographic scheme. For encryption purposes, the Java cryptography library is used. To encrypt data, we have considered three cryptographic schemes: (1) SHA-1, (2) SHA-2, and (3) Error-Tolerant Linking Algorithms. The first two schemes, SHA-1 and SHA-2, do not require any key pair, whereas the Error-Tolerant Linking Algorithms needs a key pair for encryption. As of now, we have considered that the organization and its collaborator will know the key pair beforehand. The first two schemes, SHA-1 and SHA-2, work in the same way. After encrypting the record set, party B sends the encrypted data to party A. After receiving the encrypted data from party B, party A applies the data matching technique on these two encrypted record sets.

The Error-Tolerant Linking Algorithm works in a little bit different way than the other two cryptographic schemes once the dataset name and attributes have been selected. Suppose party A has one medical record to be linked with the records held by party B. (The flow can be easily extended to the cases that party A has multiple medical records to be linked.) Party A sends the encrypted messages generated by their data record to be linked to party B. Then party B handles the encrypted messages as described in previous sections (ie, encrypting their own data record and multiplying their inverse with party A’s message) and sends the multiplied encrypted message back to party A. Party A decrypts the message and outputs the linking result. Party B moves to the next record and repeats the linking procedure. Party A does not need to encrypt their record again, but only needs to decrypt the messages sent from party B and output the linking result.

**Figure 8:**
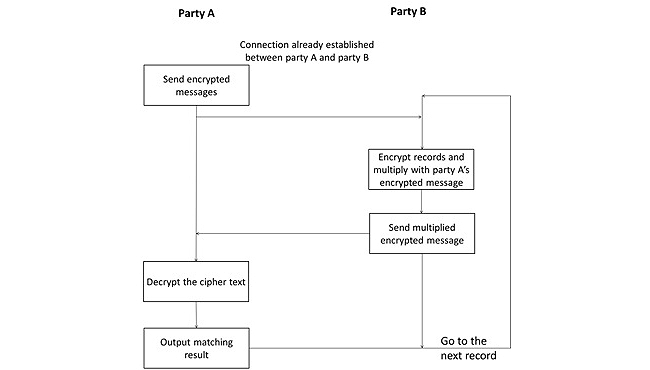
Flow of the Error-Tolerant Linking Algorithm between two parties when they are already connected and the dataset name and attributes are known to party B.

**Figure 9:**
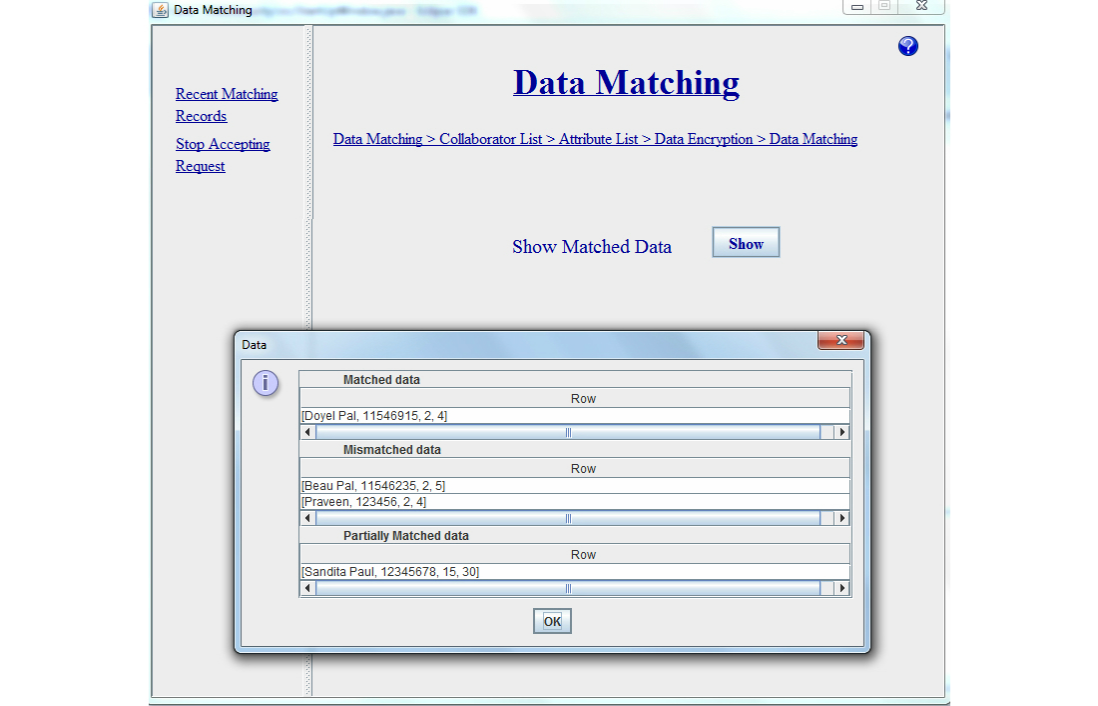
Snapshot showing the matching result in our system.

This repeating process carries on until party B has gone through all their records. Then party A and party B close the connection with each other and the privacy preserving linkage is completed. Figure 8 shows the flow of the Error-Tolerant Linking Algorithm after selecting the dataset name and attributes. Once the entire data matching procedure is completed, the client/initiating organization closes the connection with the server/participating organization automatically. As a result of the entire data matching procedure, party A gets the matched, mismatched, and partially matched result with party B. Figure 9 shows the snapshot of the matching result of our system.

### Matching Record Management Module

**Figure 10:**
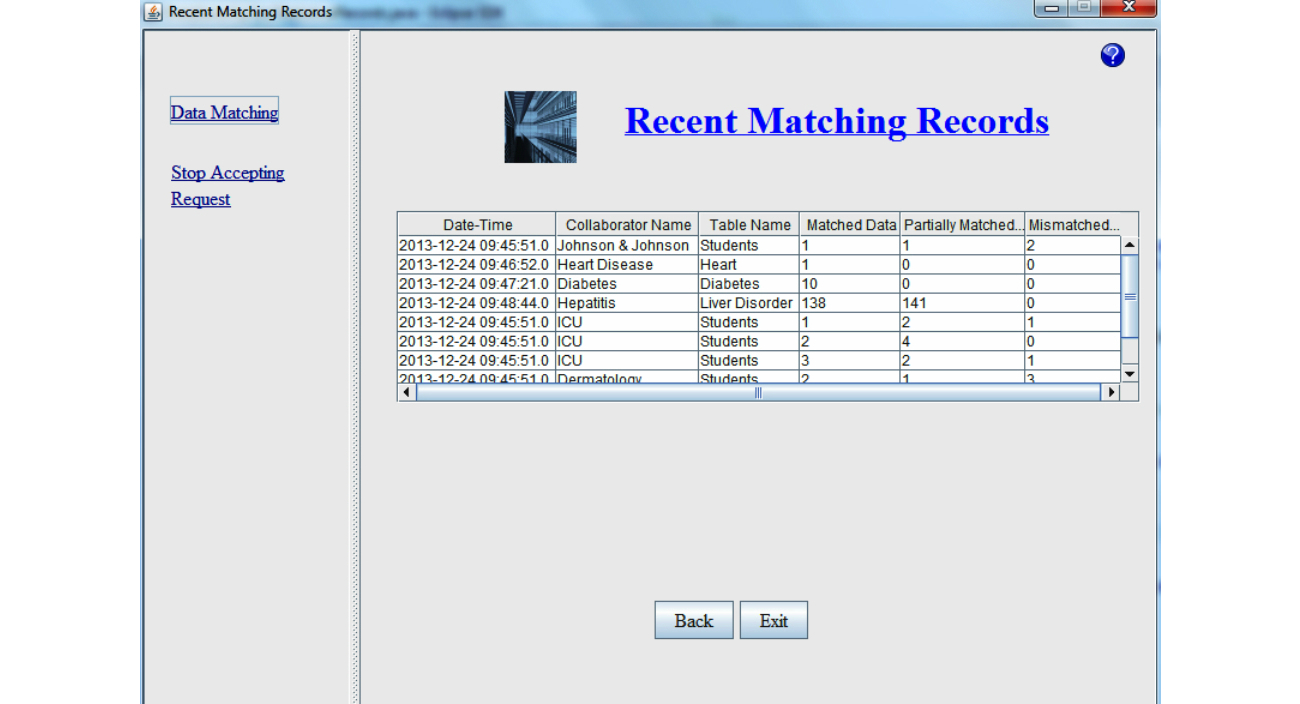
Matching Record Management Module.

The Matching Record Management Module shows the brief description of the matching result from the recent past data matching attempts. It shows the date-time of when the matching took place, name of the participating organization/collaborator, name of the record set, and the number of matched, mismatched, and partially matched data for each and every data matching attempt in table format. Figure 10 shows the snapshot of the Matching Record Management Module of our system.

### Experiment Setup

We ran our system on computers with a 3.33 GHz Intel Core i5 processor with 4 GB RAM and a 64 bit operating system. Both for party A and party B, we have constructed window applications using Java. The Internet connects the applications on different computers. The communications between party A and party B are realized by using socket API. Before running the system, each client needs to know the IP address and port number of the server. If a party/server changes their IP address, then they should inform the other parties/clients. As of now, we have considered that each party maintains an IP address, and port list of other parties.

We use two real-world medical datasets, the Pima Indians Diabetes Data Set and the Heart Disease Data Set [32] to implement our system. To handle these real-world datasets, MySQL (an open-source database system) is used. Java Database Connectivity helps to connect the application front end and the database end. It is used to access data directly from the database and to show them to the user.

### Experimental Results

We test the scalability of our system in terms of time efficiency. For each cryptographic scheme in this system, we vary the number of records and the number of attributes for each record, and then measure the computation time of our system.

To test the efficiency of our system, we consider two real-world datasets, the Pima Indians Diabetes Data Set and the Heart Disease Data Set [32]. In the Pima Indians Diabetes Data Set, we use at most eight attributes: (1) number of times pregnant, (2) plasma glucose concentration; a 2 hours in an oral glucose tolerance test, (3) diastolic blood pressure (mm Hg), (4) triceps skin fold thickness (mm), (5) 2-hours serum insulin (mm U/ml), (6) body mass index (weight in kg/height in m^2^), (7) diabetes pedigree function, and (8) age (years). Similarly for the Heart Disease Data Set, we use eight attributes for each record: (1) age of the patient, (2) sex, (3) chest pain type, (4) resting blood pressure, (5) serum cholesterol in mg/dl, (6) fasting blood sugar, (7) resting electrocardiographic results, and (8) maximum heart rate achieved. For each encryption scheme, except the Error-Tolerant Linking Algorithm, we vary only the number of attributes to four, six, and eight and use 100 patients’ records. For the Error-Tolerant Linking Algorithm, we vary the number of attributes as well as the number of patients’ records.

**Figure 11:**
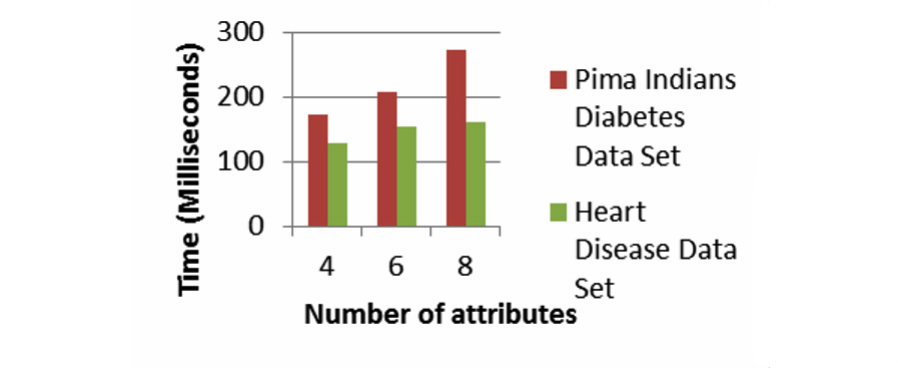
Computation time for SHA-1 for 100 records with varying number of attributes to four, six, and eight.

**Figure 12:**
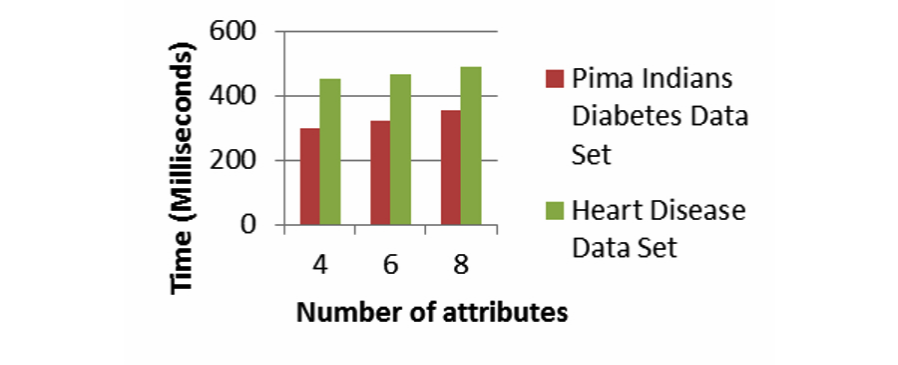
Computation time for SHA-2 for 100 records with varying number of attributes to four, six, and eight.

For the SHA-1 and SHA-2, we use 100 patients’ records from both the Pima Indians Diabetes Data Set and Heart Disease Data Set. For each record, we vary the number of attributes to four, six, and eight respectively. Figures 11 and 12 show the computation times of our system using the SHA-1 and SHA-2. For both of these above-mentioned existing algorithms, the computation time increases as we increase the number of attributes. The computation time grows almost linearly as we increase the number of attributes. Moreover, in every case the computation time does not even go beyond 0.1 second.

**Figure 13:**
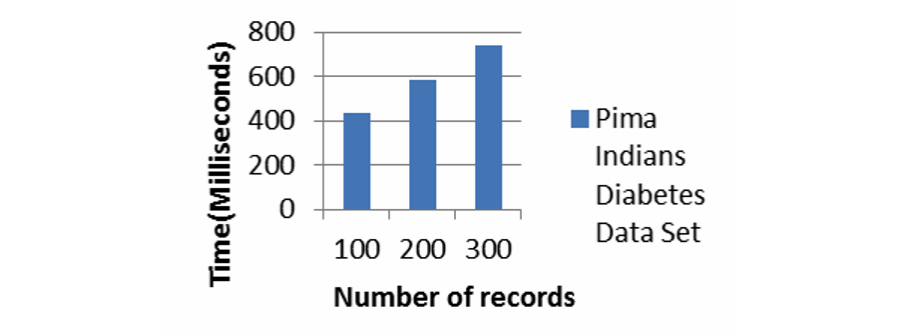
Computation time for the Error-Tolerant Linking Algorithm with varying patients’ records from Pima Indians Diabetes Data Set where each record has 4 attributes.

**Figure 14:**
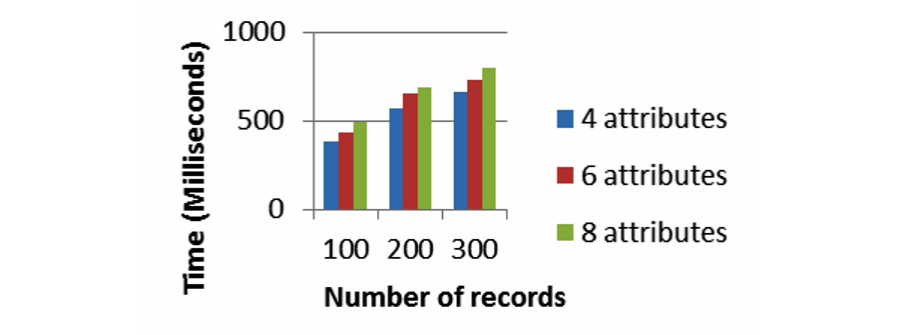
Computation time for the Error-Tolerant Linking Algorithm with varying patients’ records and varying attributes from Heart Disease Data Set.

Figures 13 and 14 show the computation time for the Error-Tolerant Linking Algorithm. We implement the Error-Tolerant Linking Algorithm with both constant and varying numbers of attributes. The values of all the attributes are preprocessed and converted to integers.

Figure 13 shows the computation time of our system when party A conducts the privacy preserving linking on the Pima Indians Diabetes Data Set. Party B holds the variable number of records varying from 100, 200, and 300, while keeping number of attributes constant as four. The computation time increases linearly as the size of data to be linked grows. Figure 14 presents the computation time for the Heart Disease Data Set with varying numbers of records and varying numbers of attributes. For this data set too, the computation time increases as the number of attributes and number of records grow. In both cases, when the number of records or number of attributes increases, the computation time increases almost linearly. In addition to that, for this algorithm too, the computation times never go beyond 0.1 second.

## Discussion

### Principal Findings

To enhance the health care quality and public health surveillance, privacy preserving medical record linkage among different medical service providers is very important. As the real-world medical record may well be error-prone, the goal of our study was to design and develop a software system that helps medical record linkage for both error-free data and error-prone data, and preserves privacy too. We have successfully designed a comprehensive system to achieve this goal. Moreover, our software meets the regulation of HIPAA and does not require a trusted third party. Our software preserves privacy since no party can get to know about another’s database. As the existing works on error-prone data are limited to textual data, we propose a novel algorithm named the Error-Tolerant Linking Algorithm, which works on error-prone numeric data. We offer two cryptographic schemes, the SHA-1 and SHA-2 for error-free data. We designed our software open and each cryptographic scheme is independent to each other so that any existing work/cryptographic scheme for error-prone textual data can be integrated later. We tested our system on real-world datasets and got the expected result each time for each of the offered cryptographic schemes. Besides that, our system is efficient for real-world datasets and the computation time for each attempt has never gone beyond 0.1 second.

### Limitations

The one limitation of our proposed system is that for error-prone data our system is limited to only numeric data. Considering this fact, we designed our software in such a way that any existing solution for error-prone textual data can be easily integrated into our system. This makes our software flexible and open to integrate any existing record linkage scheme for error-prone textual data.

### Conclusions

In this paper, we propose a solution for privacy preserving record linkage for error-free data as well as for error-prone data. For error-free data, we offer two existing cryptographic schemes: (1) SHA-1, and (2) SHA-2. A new algorithm is proposed for error-prone numeric data. We implement our system fully and tested it on two real-world data sets. We have shown that our system is secure, correct, and efficient and does not require a trusted third party. The experimental results demonstrate the efficiency of our system.

## Multimedia Appendix 1

Snapshot of selecting a function in our system.

## Multimedia Appendix 2

Snapshot of selecting a collaborator.

## Abbreviations

API: application program interface
HIPAA: Health Insurance Portability and Accountability Act
IP: Internet protocol
MD4: Message Digest Algorithm 4
NSFC: National Science Foundation of China
SHA: Secure Hash Algorithm
SMC: secure multi-party computation
SSN: Social Security Number

## References

[1] Vest JR (2009). Health information exchange and health care utilization. J Med Syst.

[2] Shapiro JS, Kannry J, Lipton M, Goldberg E, Conocenti P, Stuard S, Wyatt BM, Kuperman G (2006). Approaches to patient health information exchange and their impact on emergency medicine. Ann Emerg Med.

[3] HealthIT.gov.

[4] US Department of Health and Human Services.

[5] Bellare M, Canetti R, Krawczyk H (1996). UCSD CSE-Computer science and engineering.

[6] Chen T, Zhong S (2010). Proceedings of the AMIA.

[7] Quantin C, Bouzelat H, Dusserre L (1997). A computerized record hash coding and linkage procedure to warrant epidemiological follow-up data security. Stud Health Technol Inform.

[8] Quantin C, Bouzelat H, Allaert FA, Benhamiche AM, Faivre J, Dusserre L (1998). How to ensure data security of an epidemiological follow-up: Quality assessment of an anonymous record linkage procedure. Int J Med Inform.

[9] Alhaqbani B, Fidge C (2008). IEEE Explore Digital Library.

[10] Churches T, Christen P (2004). Some methods for blindfolded record linkage. BMC Med Inform Decis Mak.

[11] Lindell Y, Pinkas B (2000). ICT.

[12] Agrawal R, Srikant R (2000). ACM Sigmod Record 29.2.

[13] Goldreich O (2009). Volume 2-basic applications. Foundations of cryptography.

[14] Scannapieco M (2007). Privacy preserving schema and data matching. https://www.cs.purdue.edu/homes/ake/pub/main.pdf.

[15] Hjaltason GR, Samet H (2003). Properties of embedding methods for similarity searching in metric spaces. IEEE Trans. Pattern Anal. Machine Intell.

[16] Inan A, Kantarcioglu M, Bertino E, Scannapieco M (2008). Data Engineering: ICDE.

[17] Zhang W, Wang X, Lu B, Kim TH (2013). Secure encapsulation and publication of biological services in the cloud computing environment. Biomed Res Int.

[18] Gkoulalas-Divanis A, Loukides G (2012). SDM.

[19] Kum HC, Krishnamurthy A, Machanavajjhala A, Reiter MK, Ahalt S (2013). Privacy preserving interactive record linkage (PPIRL). J Am Med Inform Assoc.

[20] Mohammed N, Jiang X, Chen R, Fung BC, Ohno-Machado L (2013). Privacy-preserving heterogeneous health data sharing. J Am Med Inform Assoc.

[21] Schnell R, Bachteler T, Reiher J (2009). Privacy-preserving record linkage using Bloom filters. BMC Med Inform Decis Mak.

[22] Bachteler T, Schnell R, Reiher J (2010). Willkommen an der Universität Duisburg-Essen.

[23] Wang J, Donnan PT (2002). Journal of Applied Statistics 29.6.

[24] Weber SC, Lowe H, Das A, Ferris T (2012). A simple heuristic for blindfolded record linkage. J Am Med Inform Assoc.

[25] Shamir A, Adleman L, Rivest RL (1978). Commun. ACM.

[26] Elgamal T (1985). A public key cryptosystem and a signature scheme based on discrete logarithms. IEEE Trans. Inform. Theory.

[27] National Institute of Standards and Technology (NIST) Computer Security Division Computer Security Resource Center.

[28] Boneh D, Goh EJ, Nissim K (2005). Evaluating 2-DNF formulas on ciphertexts. Theory of cryptography.

[29] Rivest R (1992). Internet Engineering Task Force.

[30] Burr WE (2006). Cryptographic hash standards: Where do we go from here?. IEEE Secur. Privacy Mag.

[31] Boneh D (1998). Stanford.

[32] UCI Machine Learning Repository.

