# Supplementary figures and images for: Designing an Algorithm to Preserve Privacy for Medical Record Linkage With Error-Prone Data
Source: JMIR Med Inform. 2014 Jan 20;2(1):e2. doi: 10.2196/medinform.3090 (PMC4288117; doi:10.2196/medinform.3090)

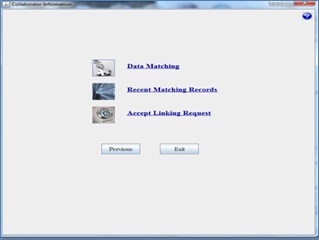

Supplement: Supplementary file 1 [file medinform_v2i1e1_app1.jpg]

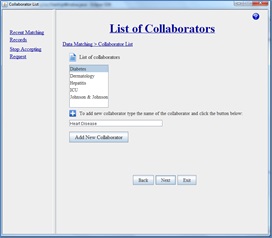

Supplement: Supplementary file 2 [file medinform_v2i1e1_app2.jpg]
